# The gut microbial metabolite imidazole propionate inhibits metformin action

**DOI:** 10.1111/jdi.13566

**Published:** 2021-05-24

**Authors:** Kenji Sugawara, Wataru Ogawa

**Affiliations:** ^1^ Division of Diabetes and Endocrinology Department of Internal Medicine Kobe University Graduate School of Medicine Kobe Japan

## Abstract

Imidazole propionate inhibits metformin action in a manner dependent on a p38γ‐Akt‐AMPK axis.

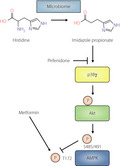

Recent attention has focused on the relation between the intestinal microbiome and metabolic diseases. Diabetes is one such disease that has been found to be influenced by changes in the composition and function of the gut microbiome. In addition, the intestinal microbiome has also been implicated in modulation of the efficacy and metabolism of certain drugs^1^. Although the mechanisms by which intestinal bacteria affect diabetes and drug efficacy remain unclear, one hypothesis is that metabolites produced by the microbiome from dietary substances play an important role. Such metabolites have been found to include short‐chain fatty acids, amino acid metabolites, trimethylamine *N*‐oxide, secondary bile acids, and polyphenol derivatives, but which of these are responsible for effects of the microbiota on the host is yet to be determined.

Metformin is one of the most widely prescribed oral antidiabetic drugs and is well known to alter the composition and metabolic profile of the gut microbiome^2^, effects that are important for its anti‐hyperglycemic and appetite‐suppressing actions. The therapeutic actions of metformin are also known to vary greatly among individuals, with genetic variation – in particular, polymorphisms in the genes for transporters such as organic cation transporter 1 (OCT1) and glucose transporter 2 (GLUT2) – having been identified as a major determinant of the response to metformin. In addition to gene polymorphisms, Koh *et al*.^3^ recently revealed that a metabolite produced by the intestinal microbiome contributes to interindividual variability in metformin response.

The same group previously performed metabolomic analyses of plasma from individuals with type 2 diabetes and healthy subjects in order to identify metabolites that might play a role in the pathology of this disease. They found that a gut bacterial metabolite of histidine, imidazole propionate (ImP) (Figure 1), was present at higher concentrations in the plasma of patients with type 2 diabetes. This metabolite was also shown to be capable of directly influencing insulin signaling and glucose tolerance^4^. In their new study, Koh *et al*.^3^ set out to examine whether ImP might also contribute to the interindividual variability in metformin response and to identify potential interactions between ImP and signaling pathways modulated by metformin.

**Figure 1:**
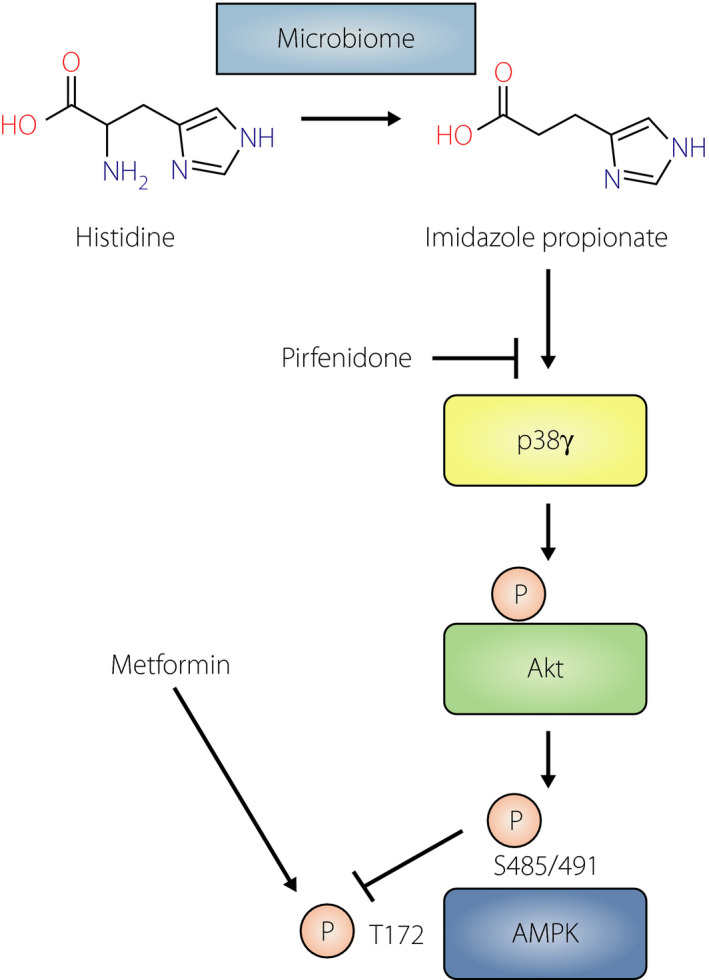
Proposed mechanism for the inhibition of metformin action by imidazole propionate (ImP) in a manner dependent on a p38γ‐Akt‐AMPK axis. ImP, a metabolite produced by the intestinal microbiome from histidine, directly activates p38γ. Akt phosphorylation by p38γ further triggers 5′ adenosine monophosphate‐activated protein kinase (AMPK) phosphorylation at S485/S491, which is involved in the inhibition of phosphorylation at T172 by metformin. Pirfenidone prevents the inhibition of metformin action by ImP.

They first divided individuals with type 2 diabetes who were taking metformin into two groups on the basis of their blood glucose levels, and they measured the concentration of ImP in plasma. They found that the ImP level was increased in the group with poor blood glucose control, suggesting that ImP might negatively affect metformin action in humans. The researchers obtained further support for this notion by showing that a single intraperitoneal injection of ImP in normal or diabetic model mice attenuated the suppressive effect of metformin on the increase in blood glucose level after glucose loading.

Koh *et al*.^3^ then sought to elucidate the molecular mechanism of the inhibitory effect of ImP on metformin action. Given that activation of 5'‐adenosine monophosphate‐activated protein kinase (AMPK) plays an important role in metformin action, they examined the effect of ImP on AMPK activity. Metformin activates AMPK in the liver by inducing its phosphorylation at threonine‐172 (T172), but administration of ImP to mice promoted phosphorylation of AMPK at serine‐485 (S485, in the α1 subunit) or serine‐491 (S491, in the α2 subunit), with such phosphorylation having previously been shown to inhibit AMPK phosphorylation at T172 and thereby to attenuate enzymatic activity. These findings thus clarified that metformin‐induced phosphorylation of AMPK at T172 is inhibited by ImP‐induced phosphorylation of the enzyme at S485/S491 (Figure 1).

The authors then focused on the protein kinase Akt to clarify the molecular mechanism of ImP action at a level upstream of AMPK, given that Akt mediates phosphorylation of AMPK at S485 and that phosphorylation (activation) of Akt increases in parallel with phosphorylation of AMPK at S485^5^. They confirmed that Akt phosphorylation in cultured HEK293 cells or in mouse tissues was increased by ImP treatment. Together with the results of additional *in vitro* analyses performed with inhibitors of Akt and other signaling inhibitors, these findings suggested that ImP‐induced inhibitory phosphorylation of AMPK *in vivo* is mediated by Akt (Figure 1).

Koh *et al*.^3^ further attempted to identify the kinase responsible for the phosphorylation of Akt induced by ImP. A search of an open‐source database implicated p38 mitogen‐activated protein kinase (MAPK) as one such candidate. Indeed, an *in vitro* kinase assay showed that the p38γ isoform of this kinase directly phosphorylated Akt in the presence of ImP. Moreover, knockdown of p38γ blocked ImP‐induced Akt and AMPK‐S485 phosphorylation in cultured cells. Together, these results suggested that a p38γ‐Akt‐AMPK axis is responsible for inhibition of metformin action by ImP (Figure 1).

Finally, the authors showed that inhibition of p38γ by pirfenidone, which is administered as a drug for idiopathic pulmonary fibrosis, prevented the inhibition of metformin action by ImP, suggesting that pirfenidone might be a candidate for combination therapy in individuals with type 2 diabetes who are not fully responsive to metformin (Figure 1).

There are some limitations to the new study of Koh and coworkers^3^. As the authors point out, given the cross‐sectional nature of the cohort of metformin‐treated subjects with type 2 diabetes, it is unclear whether those with higher blood glucose values (and higher plasma ImP levels) actually responded poorly to metformin or had more severe diabetes before treatment with the drug. A longitudinal cohort study will thus be required to determine whether ImP has a direct adverse effect on metformin action and whether metformin increases the abundance of ImP‐producing bacteria.

There also seems to be a discrepancy between the concentrations of ImP found in human plasma and those found to be effective experimentally. Whereas the average concentration of ImP in plasma is in the tens of nanomolar range, phosphorylation of AMPK in cultured cells was induced by ImP at 10–100 μM. In addition, the difference in plasma ImP concentrations between metformin‐responsive and metformin‐resistant patients was about 15–20 nM. It will thus be of interest to clarify to what extent physiological concentrations of ImP affect metformin action and whether such small differences can explain the interindividual variability in metformin action.

Despite such limitations, the findings of Koh *et al*.^3^, ^4^ have implicated a metabolite produced by the intestinal microbiome in modulation of insulin signaling and the efficacy of metformin. They have thus provided important insight into the contribution of diet‐related cross talk between the host and the microbiome to certain conditions, and further advances in this field may lead to the identification of novel drug targets. One candidate is the interaction between ImP and the adenosine triphosphate (ATP) binding pocket of p38γ. By *in silico* analysis, Koh *et al*.^3^ showed that ImP can compete with pirfenidone for binding to this site of p38γ. For structure‐based drug design, it will be desirable to elucidate the details of the mechanism by which ImP binds to p38γ and regulates its activity at the structural level. In addition to antagonism of this interaction of ImP, strategies that attenuate ImP production, such as inhibition of ImP‐producing enzymes in gut bacteria and targeting of ImP‐producing bacteria themselves, may also be attractive new therapeutic approaches for type 2 diabetes.

## DISCLOSURE

W.O. has received research support from Astellas, Dainippon‐Sumitomo Pharma, Daiichi Sankyo, Novartis, and Takeda Pharmaceutical as well as lecture fees from Astellas, Dainippon‐Sumitomo Pharma, Novartis, and Takeda Pharmaceutical. K.S. declares no conflict of interest.
